# Green Carbon Nanostructures for Functional Composite Materials

**DOI:** 10.3390/ijms23031848

**Published:** 2022-02-06

**Authors:** Ana Barra, Cláudia Nunes, Eduardo Ruiz-Hitzky, Paula Ferreira

**Affiliations:** 1 Department of Materials and Ceramic Engineering, CICECO–Aveiro Institute of Materials, University of Aveiro, 3810-193 Aveiro, Portugal; abarra@ua.pt; 2 Materials Science Institute of Madrid, CSIC, c/Sor Juana Inés de la Cruz 3, 28049 Madrid, Spain; eduardo@icmm.csic.es

**Keywords:** reduced graphene oxide, clays, hydrothermal carbons, supported carbons, polymer composites

## Abstract

Carbon nanostructures are widely used as fillers to tailor the mechanical, thermal, barrier, and electrical properties of polymeric matrices employed for a wide range of applications. Reduced graphene oxide (rGO), a carbon nanostructure from the graphene derivatives family, has been incorporated in composite materials due to its remarkable electrical conductivity, mechanical strength capacity, and low cost. Graphene oxide (GO) is typically synthesized by the improved Hummers’ method and then chemically reduced to obtain rGO. However, the chemical reduction commonly uses toxic reducing agents, such as hydrazine, being environmentally unfriendly and limiting the final application of composites. Therefore, green chemical reducing agents and synthesis methods of carbon nanostructures should be employed. This paper reviews the state of the art regarding the green chemical reduction of graphene oxide reported in the last 3 years. Moreover, alternative graphitic nanostructures, such as carbons derived from biomass and carbon nanostructures supported on clays, are pointed as eco-friendly and sustainable carbonaceous additives to engineering polymer properties in composites. Finally, the application of these carbon nanostructures in polymer composites is briefly overviewed.

## 1. Introduction

The discovery of graphene, a two-dimensional (2D) material composed of sp^2^ carbon monolayer arranged into a hexagonal network, had a tremendous impact in carbon materials research [1]. Graphene was isolated from graphite by mechanical exfoliation with an adhesive tape for the first time in 2004 by Geim et al. [2]. The relevance of this work was recognized by the attribution of the Nobel Prize in Physics in 2010. The ideal single-layer graphene has exceptional physical properties, such as ultrahigh charge-carrier mobility (200,000 cm^2^ V^−1^ s^−1^ at room temperature), high Young’s modulus (∼1.0 TPa), high specific surface area (theoretical value of 2630 m^2^ g^−1^), absorption of only 2.3% of visible light, and high thermal conductivity (∼500 W m^−1^ K^−1^). Therefore, graphene is a promising material for many distinct areas, such as energy, medicine, or electronics [3,4,5].

Eighteen years after the graphene isolation, the 2D carbon materials research is greatly developed. However, the word “graphene” has been widely misused to designate distinct 2D carbon materials when it should be reserved for the graphene sheets [1]. The intense research around graphene led to a diversification of the synthesis methods and graphene materials synthesized. Different synthesis techniques produce graphene derivatives with distinct features, varying in number of layers, lateral size, yield, type of defects, and consequently, properties [6]. 

Graphene derivatives, including graphene nanoplatelets (GNP) [7], graphene oxide (GO) [8], and reduced graphene oxide (rGO) [9], are suitable fillers for the development of polymer composites. However, synthesis challenges associated with difficulties to scale up turn the graphene derivatives expensive nanomaterials [10]. Besides, the use of toxic chemicals prevents their safe application in target areas, such as the food packaging industry and the biomedical field [11]. In this context, sustainable synthesis methods to produce green graphene derivatives avoiding toxic chemicals have been developed. The production of rGO using green chemical reductants to replace the toxic ones is a major example. Furthermore, the development of alternative carbon nanostructures using natural feedstock as carbon precursors encompasses diverse sustainable strategies to address these issues. 

This review presents different approaches to prepare sustainable carbon nanostructures suitable for polymer-based composites. The recent advances regarding the green chemical reduction of GO are overviewed. The sustainable production of hydrothermal carbon nanostructures, graphitic materials derived from biomass, and graphitic materials supported on clays are highlighted. Finally, the application of these green carbon nanostructures in polymer composites is overviewed.

## 2. Chemical Reduction of Graphene Oxide

**Figure 1:**
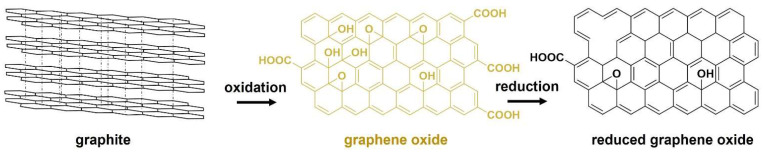
The chemical production of graphene oxide from graphite.

rGO is the most used 2D carbon material for the development of electrically conductive and mechanically reinforced polymer composites [3]. Graphite, constituted by graphene layers bonded by strong van der Waals forces, is the bulk starting material to synthesize rGO. First, graphite is oxidized to produce GO. After that, GO suffers a reduction step to produce rGO, as shown in Figure 1.

Hummers’ method and its variations are currently the most used procedures to synthesize GO [12]. The chemical exfoliation of graphite is achieved using strong acids, as concentrated sulfuric and phosphoric acids, which promote the graphene layers’ separation. The oxygenation of the separated graphene layers is accomplished using oxidants, such as hydrogen peroxide and potassium permanganate. The reduction process consists in the partial removal of oxygen functionalities present in the GO structure, namely, tertiary alcohols and epoxides attached to sp^3^ carbons, and hydroxyl and carboxylic groups attached to the sp^2^ lattice. This process converts the hydrophilic and insulator GO (yellow) into the hydrophobic and electrically conductive rGO (black) [13]. The extent of oxygen removal relies on the efficiency of the reducing agent. However, the deoxygenation is incomplete and the remnant oxygen functionalities promote the rGO dispersion and functionalization and may interact with polymers, being an advantage for the preparation of composites.

### 2.1. Evaluation of the Reduction Extent

The conversion of GO into rGO can be confirmed by different characterization techniques, such as Raman spectroscopy, X-ray photoelectron spectroscopy (XPS), and X-ray powder diffraction (XRD). Other techniques, such as Fourier-transform infrared spectroscopy (FTIR), ultraviolet–visible spectrophotometry (UV–Vis), and thermogravimetric analysis (TGA), are also useful tools. 

Raman spectroscopy is a nondestructive technique employed to evaluate the structural changes that take place upon GO-to-rGO conversion. Graphene derivatives present characteristic D and G bands at ~1355 and ~1575 cm^−1^, respectively. The D band is activated by the presence of defects such as edges, vacancies, and grain boundaries in the carbon lattice. The G band corresponds to the vibration mode of sp^2^ carbon atoms. The intensity ratio of the D band over the G band (I_D_/I_G_) is a parameter commonly used to access the level of disorder of graphene materials. The increment of sp^2^ graphitic domains and decrease in defect density may lead to a decrease in I_D_/I_G_ ratio [14]. However, the I_D_/I_G_ ratio of rGO is frequently reported to increase, which is justified if the new graphitic domains have a lower average size compared with the ones present in GO. Additionally, new defects may be introduced due to the removal of oxygen functionalities [15,16,17].

XPS is a powerful technique used to determine the surface chemical composition and the local chemical environment of the elements. The sp^2^/sp^3^ carbon hybridization is accessed through the fitting of the C 1s spectrum. The quantification of carbon and oxygen atomic percentages is used to determine the C/O ratio, which is one of the main parameters to evaluate the extent of the GO reduction [18,19]. 

XRD is used to monitor the evolution of the crystalline structure and lattice parameters upon reduction. The diffractogram of GO shows a characteristic peak at around 2ϴ = 10° corresponding to the reflection (001), with a typical d-spacing value of ~0.85 nm, caused by the presence of oxygen between GO sheets. A small peak around 2ϴ = 42° corresponding to the reflection (102) can also be observed. After the reduction, the peak at 2ϴ = 10° disappears or becomes less intense (in cases of mild reduction), and a new peak at ~2ϴ = 25° corresponding to the (002) reflection appears. The d-spacing associated with this reflection is commonly around 0.35 nm, but it depends on the extent of oxygen removal from GO. Additionally, the functionalization of rGO by the reducing agent might increment this value [20].

UV–Vis spectrophotometry can be used to monitor the reduction in case of rGO dispersions with homogeneous spatial distribution of particles. GO presents a strong absorption peak at approximately 230 nm, attributed to the π–π* transitions of aromatic C–C bonds, and a weaker peak at approximately 280 nm, assigned to the n–π* transitions of C=O bonds. After reduction, the peak at 230 nm redshifts to approximately 270 nm, suggesting the restoration of the sp^2^ graphene lattice [21,22,23].

The TGA signature of carbon materials typically presents three characteristic regions. The first region, <100 °C, is attributed to water evaporation. The second region, between 100 and 360 °C, is related to the decomposition of oxygen-containing groups. Finally, the third one, between 360 and 1000 °C, is attributed to carbon combustion when TGA is performed under air flow, or degradation of unstable carbon in case of being performed under inert atmosphere. The high percentage of oxygen functional groups on the GO structure leads to a thermal degradation at lower temperatures than rGO, which allows us to distinguish both materials by TGA [24,25].

### 2.2. Typical Reduction Methods

In the last few years, many innovative reduction strategies have been reported. The main approaches to reduce GO are chemical reduction [26], thermal decomposition [19], or strategies involving a combination of thermal and chemical methods [20]. Electrochemical reduction is another eco-friendly methodology, where GO is reduced in a standard electrochemical cell through the application of voltage. This method allows the simultaneous reduction and deposition of rGO into a substrate [27]. These approaches can produce rGO with high a C/O ratio and electrical conductivity. Nevertheless, thermal decomposition (typically above 500 °C) requires a high energy consumption, which turns difficult the scale-up process. Thus, chemical reduction is the most exploited approach for the large-scale production of rGO. 

Hydrazine is pointed as the most effective chemical reducing agent, producing rGO with a high electrical conductivity. In 2007, Stankovich et al. [15] reported the reduction of GO with hydrazine hydrate at 100 °C for 24 h. The rGO sheet aggregates presented an electrical conductivity of 2420 S m^−1^. In 2008, Li and coworkers [23] improved this reduction method, being able to produce rGO-stable water dispersions through the addition of ammonia solution to the reaction. The rGO film prepared by vacuum filtration presented an enhanced electrical conductivity of ~7200 S m^−1^. Given these results, and despite its hazardous and pollutant features, hydrazine is still used as the reference reducing agent [28,29]. 

The growing interest in rGO-based materials ignited this research field, having currently known more than 50 types of chemical reducing agents [11]. Sulfur-containing compounds [30], nitrogen-containing compounds [31], and oxygen-containing compounds [20] are categorical examples that illustrate the diverse nature of reducing agents. N- and S-containing reducing agents can simultaneously reduce GO and produce N- or S-modified rGO, with particular interest in diverse rGO applications. Wang et al. [32] synthesized S-modified rGO through the reduction of GO by cystamine dihydrochloride under basic conditions at 55 °C during 24 h. The S-modification reinforced the interactions between rGO and rubber, and consequently improved the mechanical and thermal properties of rubber-based composites in comparison with rGO reduced with hydrazine. Zhang et al. [33] reported N-modified rGO synthesized through a simple one-step hydrothermal reaction of GO in the presence of ammonium carbonate used as a chemical reductant. The N-modified rGO revealed improved electrochemical performances suitable for energy storage electrode materials.

The most effective reducing agents are commonly toxic compounds, which limit the use of rGO for food, biological, or medical applications. Therefore, many nontoxic eco-friendly reducing agents have been investigated.

### 2.3. Green Chemical Reduction

Ascorbic acid, commonly known as vitamin C, is one of the most used green reducing agents. The reduction of GO with L-ascorbic acid at room temperature during 48 h achieved an electrical conductivity of ~800 S m^−1^ [34]. Ascorbic acid also plays a role in the rGO stabilization. The oxidized ascorbic acid products can establish hydrogen bonds with the remaining oxygen-containing groups present in rGO. These interactions prevent the π–π stacking between rGO sheets, which decreases the rGO agglomeration and promotes the water dispersion. After this, many green reducing strategies have been tested. 

Table 1 lists the works, published in the last three years, regarding the green reduction of GO. These works are grouped into three main categories—plant extracts, bacteria, and combined methods—where a green chemical reducing agent is used along with a thermal or mechanical method. Plant extracts are the main category of green reducing agents due to their low cost, abundancy, and rich composition in natural reducing compounds. For example, eucalyptus bark aqueous extract was used to reduce GO, resulting in highly reduced few layer rGO. XPS analysis determined a significant increase in C/O ratio from 5.06 in GO to 10.9 in rGO, pointing an efficient oxygen removal. The reduction efficiency of this extract is attributed to the high content in polyphenolic compounds present in its composition [35]. In another work, GO was reduced with *Thuja orientalis* seed extract. Gas chromatography with a mass spectrometry detector (GC–MS) analysis pointed alpha-tocopherol as the main reducing compound [36]. *Syzygium samarangense* ripened fruit extract was used to reduce GO, producing rGO with a 4.8 C/O ratio. The ascorbic acid and aspartic acid present in this fruit composition are behind the reducing ability of the extract [37]. Similarly, the reduction of GO with *Bougainvillea glabra* flower yielded rGO with a 4.6 C/O ratio due to a rich composition in caffeic, gallic, and tannic acids [21]. 

The reduction of GO with plant extracts is usually considered a simple process. The plant extracts can be prepared from different parts of the plant, e.g., leaves, seeds, fruits, flowers, or bark, being typically prepared by reflux. After that, the reduction of GO is typically achieved by stirring the extract with a GO solution at temperatures below 100 °C between 1 and 40 h. Therefore, the reduction of GO with plant extracts is a simple and sustainable process. Given the diversity of plants with a rich composition in reducing compounds, the exploration of novel plant extracts to reduce GO is expected to continue during the next years.

Bacteria are another category of green reducing agents reported in the last 3 years, as shown Table 1. The process is also simple, avoiding toxic chemicals and high energy consumption. GO reduction is achieved through the extracellular electron transfer from the bacterial cell to GO. Bacterial reduction can be achieved by simply placing bacteria in contact with a GO solution [38,39]. Another approach is the use of bacterial polymers. Wang et al. [40] used extracellular polymeric substances extracted from *Bacillus* sp. NT 10 in the presence of ammonia to convert GO with a 1.2 C/O ratio into rGO with a 3.2 C/O ratio. The reduction was attributed to the electron-rich proteins present in the bacterial extracellular polymeric substances, along with a synergetic effect of the reducing power of ammonia ions. Eco-friendly sulfur and nitrogen rGO modification can also be accomplished using bacteria. Dong et al. [41] reported a use of *Desulfotomaculum* sulfate-reducing bacteria to produce N- and S-modified rGO. A GO film was prepared by solvent casting and incubated with bacteria for a few days at 37 °C. The resulting material showed a 4.5 C/O ratio and 3.71% and 0.72% atomic percentages of nitrogen and sulfur heteroatoms, respectively. Similarly, Kalathil et al. [38] reduced GO with *Geobacter sulfurreducens* and acetate. The *Geobacter*/rGO material showed a 5.5 C/O ratio and heteroatom modification with 5% N and <1% of S, P, Fe, and Cu. 

The combination of chemical and physical reduction methods is a strategy to improve the extension of reduction while maintaining mild conditions, as shown in Table 1. Recently, we reported the synthesis of rGO by the hydrothermal treatment of GO in the presence of caffeic acid. Caffeic acid not only reduced rGO, but also produced carbon particles that can be used for further rGO functionalization [20]. Furthermore, ZnO nanostructures were grown in situ with simultaneous reduction of GO sheets by a solvothermal method using ethanol as solvent. The obtained ZnO–rGO nanostructures can be used as functional fillers due to the antimicrobial activity of ZnO [42]. Narayanan et al. [25] also reported the hydrothermal synthesis of rGO using starch as reducing agent. The combination of the green chemical reduction with mechanical exfoliation (e.g., Taylor vortex flow or ball milling) is another eco-friendly strategy to synthesize rGO [43,44]. The green reduction of GO is a low-cost process. The selection of nonhazardous reducing agents commonly produces nontoxic and biocompatible rGO suitable to be used in biomedical or food applications [45,46]. For example, the N- and S-modified rGO film prepared by bacterial reduction was directly used to cultivate MCF−7 breast cancer cells on top, being used as an electrochemical H_2_O_2_ sensor [41]. Similarly, the starch-modified rGO was biocompatible to human skin fibroblasts and hemocompatible to red blood cells [25]. In addition, the simultaneous reduction and modification of rGO, or even the presence of the reducing agent in the final material, might be beneficial to establish chemical interactions with polymeric matrices in the preparation of composite materials. Therefore, the green rGO has promising features for the development of polymer composites avoiding toxic compounds that compromise the biological areas.

**Table 1:** Green chemical reducing agents used to convert GO into rGO reported in the last three years.

Reductant	Conditions	C/O ratio ^a^	I_D_/I_G_	d-Spacing (nm) ^b^	Ref.
Elemental sulfur	4 h, 170 °C	13.2	0.97	0.363	[47]
POM (SiW_12_O_40_^5−^)	1 min	6.1	1.13	–	[48]
**Plant Extracts**					
*Urtica dioica* leaf	pH 12, 1 h, 90 °C	4.8	1.13	–	[29]
*Thuja orientalis* seed	6 h, RT	–	0.14	0.355	[36]
*Peganum harmala* seed	1 h, 90 °C	–	0.94	0.355	[49]
	pH 12, 1 h, 90 °C	–	0.90	0.380	[50]
*Syzygium samarangense* fruit	40 h, 60 °C	4.8	1.17	0.370	[37]
*Tridax procumbens* leaf	12 h, 95 °C	–	1.00	0.360	[45]
Gooseberry fruit	3 h, 95 °C	–	1.11	0.368	[51]
*Erythrina senegalensis* leaf	3 h, 95 °C	6.2	–	0.330	[46]
*Bougainvillea glabra* flower	5 h, 95 °C	4.6	–	0.380	[21]
Eucalyptus bark	24 h, 85 °C	10.9	1.15	0.356	[35]
*Capsicum annuum* fruit	8 h, 80 °C	–	1.30	0.341	[52]
*Camellia sinensis* leaf	pH 9, 2 h, 120 °C	–	1.14	0.337	[53]
**Bacteria**					
*Pseudoalteromonas* sp.	24 h	–	1.30	0.335	[39]
*Desulfotomaculum*	Few days, 37 °C	4.5	1.37	0.370	[41]
*Bacillus* sp. EPS	pH 8, 24 h 40 °C	3.2	1.02	0.365	[40]
*G. sulfurreducens*/acetate	48 h, 30 °C	5.5	1.18	–	[38]
*Bacillus sphaericus*	48 h, 30 °C	2.6	1.17	0.870	[54]
**Combined Methods**					
HTC/caffeic acid	24 h, 180 °C	6.0	1.09	0.343	[20]
HTC/ZnO	pH 1, 24 h, 150 °C	–	1.32	–	[42]
HTC/starch	pH 9, 15 min, 120 °C	3.6	1.03	0.378	[25]
HTC/*P. amboinicus* leaf	12 h, 120 °C	–	1.30	0.360	[55]
TVF/ascorbic acid	pH 10, 0.5 h, 95 °C	6.2	1.32	0.390	[43]
BM/Zn	6 h, RT	8.9	1.32	–	[44]

POM: polyoxometalate. RT: room temperature. EPS: extracellular polymeric substances. HTC: hydrothermal carbonization. UV: ultraviolet. TVF: Taylor vortex flow. BM: ball milling. ^a^ Values determined from XPS elemental analysis. ^b^ Values determined from XRD analysis.

## 3. Carbon Structures Derived from Biomass

Biomass typically presents a carbon content between 45% and 50%. The isolation of carbon from other chemical elements is accomplished by thermochemical treatments, such as pyrolysis, hydrothermal carbonization (HTC), or a combination of both processes. The materials obtained from biomass conversion present distinct properties that mainly rely on the starting carbon precursor and processing strategies [56,57]. 

Pyrolysis is the decomposition of biomass in temperatures typically between 350 and 1100 °C under inert atmosphere. Conventional pyrolysis is performed in a tubular furnace, where the heat generated by electricity is transferred to the biomass. The alternative microwave-assisted pyrolysis generates localized heat, being an energy efficient and time-saving method [58]. This method improves the surface area of materials. For example, hay-derived activated biochar produced by microwave pyrolysis showed a surface area 30% higher in comparison to conventional pyrolysis [59].

Starbon^®^ is a patented technology that uses conventional pyrolysis to convert polysaccharides into carbonaceous mesoporous materials, commercially designated as Starbons [60]. Starch was the first polysaccharide used as a precursor for Starbons technology. The conventional preparation route comprises several sequential steps: (i) starch gelatinization, (ii) starch retrogradation, (iii) solvent exchange, (iv) drying, and (v) carbonization. For example, corn starch was gelatinized in heated distilled water and recrystallized by cooling down at 5 °C. Water from the retrograded starch gel was removed by solvent exchange with ethanol and dried to prevent collapse of the structure. The resulting material was pyrolyzed between 150 and 700 °C, after being treated with *p*-toluene sulfonic acid to catalyze the carbonization and keep the porous structure. The expanded starch showed a Brunauer–Emmett–Teller specific surface area (S_BET_) of approximately 180 m^2^ g^−1^ and a narrow pore volume of 0.4–0.6 cm^3^ g^−1^. The hydrophobicity of these materials was controlled by the degree of carbonization, producing more hydrophilic materials at lower temperatures [61]. The application of Starbons technology to alginic acid kept the first four steps in agreement with the starch processing, but the alginic acid gel was dried with supercritical CO_2_ and pyrolyzed in a broader temperature range of 200–1000 °C. The mesoporous materials showed a S_BET_ of 200 m^2^ g^−1^, but the different pyrolysis temperatures did not influence the specific surface area. Nevertheless, the higher temperatures produced carbon materials with more graphitic domains, as demonstrated by an increment C/O ratio from XPS and elemental analysis techniques [62].

HTC is a thermochemical conversion method alternative to pyrolysis, in which biomass is processed inside a sealed autoclave at mild conditions using water as solvent. The mild processing temperatures, typically between 120 and 280 °C, generate supercritical water that promotes the biomass conversion. HTC consumes less energy compared with pyrolysis, being more sustainable from an ecological point of view. In this context, the microwave-assisted HTC is an alternative way that saves even more time and energy. For example, spherical carbon particles with a carbon content >90% were prepared by processing glucose only during 15 min by microwave-assisted HTC [63]. 

**Figure 2:**
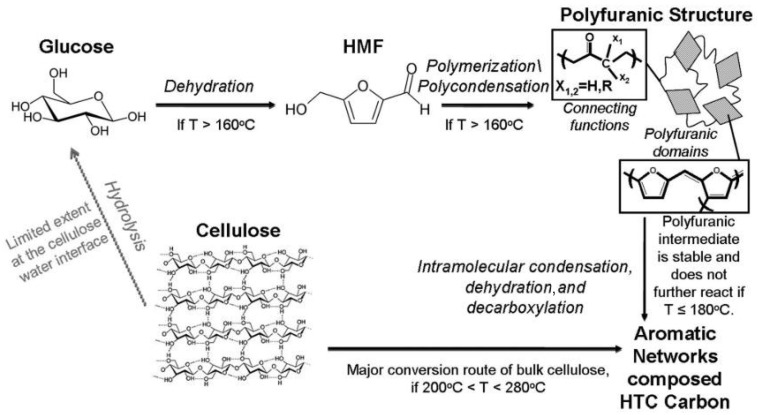
Proposed mechanism for HTC of cellulose. Reproduced with permission from Falco et al. ***Green Chem. 2011, 13, 3273*** published by the Royal Society of Chemistry [64].

The HTC of carbohydrates involves complex chemical reactions, which can be divided into five general stages: (i) hydrolysis, (ii) dehydration, (iii) decarboxylation, (iv) polymerization, and (v) aromatization [57]. Figure 2 shows the proposed mechanism for the HTC of cellulose. 

Titirici et al. [65] investigated the structure and morphology of materials processed by HTC at 180 °C during 24 h using different mono- and polysaccharides as carbon sources. Hexoses-containing compounds (glucose, maltose, sucrose, amylopectin, and starch) and hexose derivative 5-hydroxymethyl-furfural-1-aldehyde produced interconnected particles and agglomerated spheres. The hexoses dehydrate into hydroxymethyl furfural and condense to form carbonaceous materials with a similar structure and composition. The interconnected hexose-derived structures result from the good water solubility of hydroxymethyl furfural. On the other hand, xylose and furfural, a pentose-containing compound and a pentose derivative, respectively, produced well-dispersed spheres. Xylose dehydrates to form furfural, which has a limited water solubility, and polymerizes, forming carbon structures identical to the ones obtained from pure furfural. The materials obtained from mono- and polysaccharides were identical. For example, cellulose at the water/cellulose interface hydrolyzes to glucose, following the mechanism of hexoses, as shown in Figure 2. On the other hand, raw cellulose follows a reaction mechanism associated with pyrolysis yielding highly aromatic materials even at mild conditions, since high pressure destabilizes the cellulose structure. The same study compares the temperature of biomass decomposition by HTC and pyrolysis processes. Rye straw biomass submitted to HTC decomposed between 240 and 280 °C, while during pyrolysis decomposition only started at 350 °C. The lower temperature required for rye straw decomposition by HTC is attributed to the high pressure involved in the process. Another advantage of the HTC process is the possibility to control the chemical composition of carbonaceous materials such as furan-to-arene ratio [66]. 

The hydrothermal treatment introduces oxygen-containing groups to the carbon structures, typically producing materials with reduced electrical conductivity. The addition of GO to glucose, used as carbon source, before HTC increases the electrical conductivity of the hydrothermal species obtained [67]. In this context, the HTC followed by a pyrolysis step is an efficient strategy to improve the electrical conductivity, as demonstrated with the conversion of sugar cane into an electrically conductive aerogel. The aerogel conductivity increased from 0.4 to 1.3 S cm^−1^, with the increment of HTC time before pyrolysis [68]. Similarly, chitosan treated by HTC followed by pyrolysis produced carbon structures with high electrical conductivity, having the advantage of maintaining the nitrogen atoms available for further functionalization. Post-pyrolysis transforms the sp^3^ hybridized carbons into sp^2^ carbons, being a fundamental step to increase the graphitization of the hydrothermal carbons [69]. 

HTC produces carbonaceous materials with a very low surface area and undeveloped porosity. Zhong et al. [70] proposed a vapor-phase alternative HTC treatment to carbonize monosaccharides. Sucrose was treated in a glass vial placed inside an autoclave, while the gap between the autoclave and the vial was filled with water during 24 h at 200 °C. This strategy produced spongelike mesoporous carbons, in opposition to the nonporous carbon material typically obtained by conventional HTC of sucrose. The combination of HTC followed by pyrolysis also creates porosity, reinforcing the benefits of using both thermochemical processes [71,72]. In this regard, the use of templates to shape the carbon materials during biomass conversion is a powerful method to tune the porosity and surface area.

The carbon nanostructures derived from biomass are sustainable and low-cost alternatives to graphene derivatives. HTC is an economical and eco-friendly technique since it uses mild temperatures, self-generated pressure, and water as solvent. Pyrolysis uses higher temperatures in comparison with HTC; however, it can still be considered a relatively economical technique. The combination of HTC and pyrolysis techniques may lower the energy consumption required for pyrolysis, making the process more economical and eco-friendlier. The porosity and mechanical and electrical properties of these graphitic structures can be modified by the processing conditions, tailoring their properties to become fillers of polymeric composite materials.

## 4. Graphitic Materials Supported on Lamellar Structures

Clay minerals are natural and abundant resources, adequate for the sustainable development of ecological materials. The porosity and functional groups present in natural or synthetic clays turn them into suitable platforms to adsorb diverse types of molecules [73,74], leading to a wide variety of uses, including hybrid materials for advanced applications [75]. Therefore, clays have been used as porous templates to produce nanostructured carbon materials. In this context, the use of clays can be done in two different approaches: as molds or templates that are removed after carbonization of a carbon precursor [76,77] or, alternatively, as supports maintained after nanocomposite synthesis [78,79].

Sepiolite is a natural hydrated magnesium silicate showing a microfibrous morphology that has been deeply investigated to prepare carbon nanomaterials and nanocomposites as either template or support. The structure of this clay mineral is organized in alternate Mg–silicate blocks and intracrystalline nanopores aligned in the fiber direction. This structural organization, forming interior cavities (tunnels) and exterior channels, turns sepiolite into an attractive template [80]. Acrylonitrile was adsorbed into sepiolite pores, polymerized to obtain polyacrylonitrile, and thermally treated by pyrolysis at 750 °C under N_2_ flow. The resulting carbon–clay nanocomposites were electrically conductive, maintaining the silicate template, which was removed with acid treatments, and free carbon fibers with 1 µm length and 20 nm diameter were obtained [73]. Carbon–sepiolite nanocomposites derived from cellulose were synthesized by HTC using sepiolite pretreated with hydrochloric acid. These acid treatments increase the amount of surface silanol groups (Si–OH) present at the clay surface due to the extraction of Mg^2+^ ions from its structure. The resulting carbon–sepiolite nanocomposites showed an increased adsorption capacity towards organic compounds, such as methylene blue and phenols, in comparison with pristine sepiolite [81]. However, these treatments could introduce deep alterations in the crystal order of the starting sepiolite generating silica-based materials [82,83,84]. Given that sepiolite interacts with diverse biopolymers through hydrogen bonding [81,85,86], according to Wu et al. [74] the increase in acid pretreatment that increased the silanol group density promotes the interactions with cellulose and, consequently, the carbon content in the final nanocomposites [77]. However, an alternative explanation should be considered, taking into account the significant increase in the specific surface area of the silicate-based materials produced by the acid treatments, which can promote the interaction with the polymers.

Graphene-like materials were also prepared using sucrose or gelatin supported on sepiolite clay. The carbon–clay bionanocomposites obtained after pyrolysis at 800 °C under N_2_ atmosphere presented an electrical conductivity in the range of 0.01–1 S cm^−1^. The use of gelatin biopolymer as carbon precursor produced N-modified materials, being advantageous for further functionalization [78]. 

A 2D-layered silicate montmorillonite has also successfully been used as a porous template. Electrically conductive and porous caramel–clay nanocomposites were prepared from sucrose intercalated into montmorillonite in a melting process (i.e., through in situ formed caramel) [77]. In this way, the precursors in the absence of solvents were polymerized using microwave radiation and further pyrolyzed at 750 °C under N_2_ atmosphere [79]. Following this work, a water solution of caramel (commercial liquid caramel) was also used as a precursor. Caramel was impregnated into montmorillonite or sepiolite clays and thermally treated under the same pyrolysis conditions to obtain the graphene-like materials [79].

**Figure 3:**
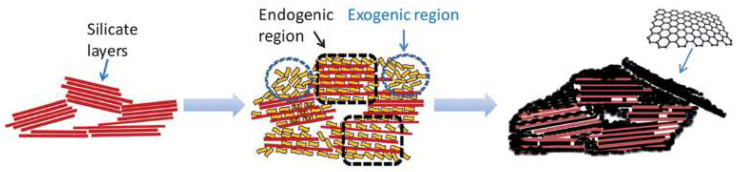
Schematic representation of graphene-like materials formed inside sepiolite pores (endogenic regions) and epitaxially grown on the sepiolite surface (exogenic regions). Reproduced with permission from Ruiz-García et al., ***J. Mater. Chem. A 2017, 2009***, published by the Royal Society of Chemistry [88].

In contrast to 2D clay minerals, such as montmorillonite, sepiolite does not have swelling properties and the formation of intercalated compounds is not possible. In this case, caramel is presumed to fill the sepiolite pores and cover the external surface in agreement with N_2_ adsorption isotherms of the resulting nanocomposites [87]. The formation of graphitic material into porous silicate templates is represented in Figure 3. The graphitic material can be formed in the interior of the pores by an endogenic mechanism or at the silicate surface by an exogenic mechanism [88]. Al-pillared montmorillonite and glucose were treated by HTC followed by pyrolysis to synthesize another family of carbon–clay nanocomposites. The thermal treatments converted glucose into carbon clusters located in the montmorillonite layers and surface. Free carbon microspheres were also formed due to the HTC process. The montmorillonite pillaring strategy improved the S_BET_ from 27.1 m^2^ g^−1^ to 129.6 m^2^ g^−1^ due to an increase in montmorillonite layers’ separation. The introduction of carbon and its conversion resulted into nanocomposites with a S_BET_ of 162.6 m^2^ g^−1^, a pore volume inferior to 0.1 cm^3^ g^−1^, and an average pore size of 4.3 nm [89]. These S_BET_ and pore volume are lower compared with the values obtained for mesoporous carbons prepared by the removal of a laponite clay template [90]. 

The removal of a clay template is advantageous to produce carbon nanomaterials with a large surface area. However, this strategy may be time-consuming and nonsustainable due to the use of toxic chemicals [77,90]. On the other hand, the maintenance of the clay template results in carbon–clay hybrid nanocomposites. The incorporation of these materials as fillers into insulating polymer matrices to produce composites can make them electrically conductive and improve their barrier and mechanical properties. The template maintenance is also advantageous for further material functionalization due to the presence of clay functional groups [87,88,91]. Nevertheless, despite the advantages of this strategy, it produces materials with inferior textural properties compared with the clay template removal [89,90].

**Figure 4:**
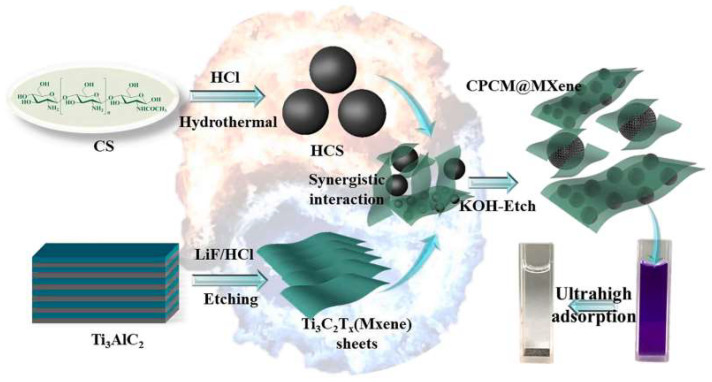
Schematic representation of the assembly of hydrothermal carbon spheres (HCS) from chitosan (CS) and Ti_3_C_2_T_x_ MXene. Reproduced with permission from Wu et al., ***Chem. Eng. J. 2021, 426, 130776***, published by Elsevier [93].

MXenes are a large family of transition metal carbides, carbonitrides, and nitrides showing a general formula, M_n+1_X_n_T_x_, where M is an early transition metal (e.g., Ti), X is carbon and/or nitrogen, and T_x_ represents termination groups (e.g., OH) [92]. Interestingly, they exhibit colloidal and surface properties in close relation to clay minerals but show useful additional properties, such as metallic electrical conductivity. These materials are excellent candidates to form carbon-based nanocomposites and, for instance, porous carbon nanospheres generated by pyrolysis of chitosan could be assembled to Ti_3_C_2_T_x_ MXene, as recently reported [93]. The resulting carbon-nanostructured materials (Figure 4) provide an elevated specific surface area (>1800 m^2^ g^−1^) and improved adsorption properties tested in dye adsorption from aqueous solutions. For instance, the adsorption capacity of crystal violet is close to 2750 mg g^−1^, which appears to be the highest adsorbed amount of dye per mass unit never reported for carbon-based materials [93].

## 5. Polymer Composites Containing rGO

Carbon nanostructures are used to reinforce the mechanical, electrical, thermal, and optical properties of polymeric matrices. Therefore, biocomposites containing carbonaceous materials have application in many distinct fields. To the best of the author’s knowledge, among the carbon nanostructures reviewed in this paper, only rGO was applied to prepare polymer-based composites. However, the carbons derived from biomass and the clay-supported carbons have potential for the development of polymer-based composites. One advantage is the selection of the carbon precursor according to the applications. For example, a carbon precursor containing N- or S- functional groups may not only reinforce the composite through the establishment of interactions with the polymer but also improve their performance on the application. Similarly, the electrical conductivity and porosity of the carbon nanostructures can be tailored by an appropriate selection of the methodology. Therefore, the alternative green carbon nanostructures are promising materials for the development of polymer-based composites.

Table 2 presents the polymer composite materials prepared using the rGO reduced by the green methodologies listed in Table 1. The applications found for these materials were corrosion protection [29,49,50], gas diffusion barriers [47], sensing [21], supercapacitors [35], environmental remediation [39], and food packaging [9,94]. Recently, we revised the use of graphene derivatives in biopolymer composite nanostructures for food packaging applications, which is an example of application where these green carbon nanostructures can be employed [95].

The mechanical reinforcement promoted by the incorporation of a carbon nanostructure into a polymer matrix is a general effect with interest in most of areas [9,44,47,50]. The synthesis methods and precursors of carbon nanostructures should be carefully selected since they can influence the preparation of composites. In some cases, the use of green chemical reductants was an advantage in the preparation of polymer composites. For example, the *Peganum harmala* seed extract used to prepare rGO had a dual role as reducing agent and corrosion inhibitor. Therefore, the coatings containing rGO prepared with this plant extract acted not only as a barrier but also as an active corrosion coating [49]. Similarly, the simultaneous reduction and modification of GO with elemental sulfur produced S-rGO with good adsorption capacities towards Hg(II) due to the high affinity between sulfur and metallic adsorbates. In addition, S-rGO showed good interfacial interactions with the polystyrene polymer, which improved the dispersibility of the filler and the mechanical properties of the resulting composites [47]. Additionally, the presence of clays used to support carbon nanomaterials can improve their dispersion into the polymer matrix [96].

## 6. Conclusions and Future Perspectives

Green carbon structures prepared using low-cost, eco-friendly, and sustainable methodologies may be additives of interest to modify the mechanical, electrical, and barrier properties of polymers. The use of natural carbon precursors, green reactants, and sustainable methodologies allows the development of versatile composites for a variety of applications in a wide number of fields, including biological and food areas. The use of carbon nanomaterials derived from biomass, such as hydrothermal carbons or carbons supported on clays, in the fabrication of polymer composites has a high potential of exploration since they were not used for this purpose yet. In addition, natural biopolymers can be used as a composite’s matrices to ensure the final material sustainability. These materials are expected to present comparable properties with the polymer-based composites using rGO as a filler, with the advantage of being cost-effective, safe, and environmentally sustainable. 

## Figures and Tables

**Table 2:** Applications of polymer-based composites prepared using the green carbon nanostructures previously reviewed.

Application	Carbon Nanostructure	Polymer Composite	Results	Ref.
Corrosion protection	rGO (*Urtica dioica* leaf)	Polyurethane/rGO (0.15 wt%) coatings (tested on mild steel)	Resistance against accelerated weathering condition; improved UV shielding and corrosion protection efficiency.	[29]
Corrosion protection	rGO (*Peganum harmala* seed)	Epoxy resin/rGO-Zn (0.15 wt%) coatings (tested on steel)	Dual active and barrier corrosion protection.	[49]
Corrosion protection	rGO (*Peganum harmala* seed)	Epoxy ester resin/rGO-Zn (0.15 wt%) coating (tested on steel)	Improved tensile strength (78%), Young’s modulus (102%) and fracture energy (83%); improved thermal stability (62%).	[50]
Gas diffusion barrier	rGO (elemental sulfur)	Polyimide/rGO (0.5–5 wt%) films	Improved tensile strength and Young’s modulus; 95% reduction of oxygen permeability.	[47]
Sensing	rGO (*Bougainvillea glabra* flower)	Nafion/rGO solution drop-casted on a carbon working electrode	Sensor electrode used for Pb^2+^ detection; improved sensitivity and ultralow limit of detection.	[21]
Supercapacitors	rGO (eucalyptus bark)	Nafion/rGO solution drop-casted on a glassy carbon electrode	High specific capacitance (239 F g^−1^) and high energy density (71 W h kg^−1^) at a current density of 2 A g^−1^.	[35]
Environmental remediation	rGO (*Pseudoalteromonas* sp.)	Sodium alginate/rGO solution dripped into CaCl_2_ solution to obtain spheres	MB and CR dye adsorption from water. Reusable absorbent with adsorption efficiency of the MB and CR 77.91% and 68.27% after 4 adsorption–desorption cycles.	[39]
Food packaging	rGO (HTC/caffeic acid)	Chitosan/rGO (50%) film	Electrically conductive film to sterilize food by in-pack PEF; electrical conductivity of 0.7 S m^−1^ and 2.1 × 10^−5^ S m^−1^ in-plane and through-plane, respectively.	[9]
Food packaging	rGO (HTC/ZnO)	Alginate/sepiolite/ZnO-rGO (50%)	Antimicrobial and electrically conductive film for food packaging. *E. coli* and *S*. Inhibition of *aureus* growth; electrical conductivity of 0.1 S m^−1^ and 7.5 × 10^−5^ S m^−1^ in-plane and through-plane, respectively.	[94]
Not mentioned	rGO (BM/Zn)	Epoxy resin/rGO (0.1–0.3%) composites	Improvement of thermomechanical properties.	[44]

rGO: reduced graphene oxide. UV: ultraviolet. MB: methylene blue. CR: Congo red. HTC: hydrothermal carbonization. PEF: pulsed electric field. BM: ball milling.
